# Antiviral Cyanometabolites—A Review

**DOI:** 10.3390/biom11030474

**Published:** 2021-03-22

**Authors:** Hanna Mazur-Marzec, Marta Cegłowska, Robert Konkel, Krzysztof Pyrć

**Affiliations:** 1 Division of Marine Biotechnology, University of Gdańsk, Marszałka J. Piłsudskiego 46, PL-81-378 Gdynia, Poland; robert.konkel@phdstud.ug.edu.pl; 2 Institute of Oceanology, Polish Academy of Science, Powstańców Warszawy 55, PL-81-712 Sopot, Poland; mceglowska@iopan.pl; 3 Virogenetics Laboratory of Virology, Malopolska Centre of Biotechnology, Jagiellonian University, Gronostajowa 7A, PL-30-387 Krakow, Poland; k.a.pyrc@uj.edu.pl

**Keywords:** cyanobacteria, antiviral metabolites, lectins, polysaccharides

## Abstract

Global processes, such as climate change, frequent and distant travelling and population growth, increase the risk of viral infection spread. Unfortunately, the number of effective and accessible medicines for the prevention and treatment of these infections is limited. Therefore, in recent years, efforts have been intensified to develop new antiviral medicines or vaccines. In this review article, the structure and activity of the most promising antiviral cyanobacterial products are presented. The antiviral cyanometabolites are mainly active against the human immunodeficiency virus (HIV) and other enveloped viruses such as herpes simplex virus (HSV), Ebola or the influenza viruses. The majority of the metabolites are classified as lectins, monomeric or dimeric proteins with unique amino acid sequences. They all show activity at the nanomolar range but differ in carbohydrate specificity and recognize a different epitope on high mannose oligosaccharides. The cyanobacterial lectins include cyanovirin-N (CV-N), scytovirin (SVN), microvirin (MVN), *Microcystis* *viridis* lectin (MVL), and *Oscillatoria agardhii* agglutinin (OAA). Cyanobacterial polysaccharides, peptides, and other metabolites also have potential to be used as antiviral drugs. The sulfated polysaccharide, calcium spirulan (CA-SP), inhibited infection by enveloped viruses, stimulated the immune system’s response, and showed antitumor activity. Microginins, the linear peptides, inhibit angiotensin-converting enzyme (ACE), therefore, their use in the treatment of COVID-19 patients with injury of the ACE2 expressing organs is considered. In addition, many cyanobacterial extracts were revealed to have antiviral activities, but the active agents have not been identified. This fact provides a good basis for further studies on the therapeutic potential of these microorganisms.

## 1. Introduction

### Viruses and Viral Infections—A Global Problem

Viruses are obligatory parasites composed of nucleic acids (DNA or RNA) packed in a protein capsid, in some cases enveloped with a lipid bilayer. The great diversity of viral species makes it difficult to unequivocally classify them into related groups. For that reason, several ‘general’ classification systems were introduced [1,2]. With respect to the genetic material, viruses can be classified as DNA, RNA, or RNA/DNA viruses. Other classifications are based on morphological features and include enveloped and non-enveloped viruses, e.g., the Baltimore classification sorts viruses into groups based on the RNA production manner [2,3].

In the 21st century, the emergence of several viral species has been observed in humans, including Ebola, Zika, Middle East respiratory syndrome (MERS), severe acute respiratory syndrome (SARS), influenza, and Nipah virus [4,5]. The majority of these species emerge in humans due to the zoonotic events, when animal viruses unknown to our immune system cross the species border and manage to adapt to the new host [6]. In some cases, the geographic distribution of these species is limited to restricted areas (e.g., MERS-CoV), while other infections (e.g., influenza) spread throughout the globe [6,7,8,9]. With global processes, such as climate change, frequent and distant travelling, rapid population growth, and substandard sanitation as well as interactions with animals and transfer of insect vectors into new areas, there is an increased risk for transmission of viral infections. For example, the *Aedes* mosquitoes played a key role in the spread of dengue virus (DENV) epidemic events in tropical and subtropical regions of Asia (70%), Africa, the Americas, and Oceania [10]. The number of DENV infections that occur annually was estimated to be 390 million [11]. The *Aedes* mosquito also transmits such infections as Chikungunya virus, Zika virus (ZIKV), Yellow Fever virus (YFV) and Rift Valley fever virus (RVFV) [12]. 

The management of viral infections, while lacking effective prevention or mitigation tools, generates costs and has serious impacts on the economy and social life. The recent COVID-19 pandemic affected all economic sectors, the health system, education, social mobility, sport, and many other areas of human activities [13]. Unfortunately, effective protection by vaccinations is available only for selected viral species [14,15]. Further, the development of effective drugs is a long and tedious process that frequently ends up being a failure. In 2016, the list of antiviral agents contained only 90 approved drugs for the treatment of 22 human infectious diseases [16]. The low number of developed drugs is linked with the aforementioned diversity of viral species, and consequent lack of broad-spectrum antivirals, as seen for the bacteria. The rapid generation of the escape mutants further hampers the process. 

Existing antiviral therapies target different steps of viral infection, from adsorption and penetration of the host cell, through uncoating and replication of nucleic acids, to viral assembly and release [17]. Unfortunately, as viral machinery is tightly fitted to the cellular microenvironment, antiviral agents often not only inhibit the viral infection but also affect the host metabolic processes. There are many other issues to be overcome in the development of effective antiviral therapeutics. These include the variable nature of viral genomes (high rate of mutation and recombination of RNA viruses), yielding rapid development of resistance towards currently used antiviral agents [18].

The most effective way to rapidly identify antiviral compounds is a high-throughput screening of libraries containing existing drugs or random molecules. Such an analysis yields potential drug candidates, but may also provide valuable data for, e.g., QSAR (quantitative structure-activity relationship) lead optimization. However, these efforts are usually cost-ineffective and limited by the available compound or fragment libraries. In the search for new antivirals, the natural products, with their diverse and unique structures and mechanisms of action, have always constituted an important source of inspiration. The nucleoside spongouridine produced by the marine sponges *Cryptotethya crypta* is one of the examples [19]. A synthetic analogue of the compound, vidarabine, as an approved drug, inhibits the replication of viral DNA, and is effective in the treatment of herpes simplex virus (HSV-1 and HSV-2) and varicella zoster virus (VZV) [20]. Natural products, such as flavonoids, oligostibens, coumarins and diarylheptanoids, are active against influenza virus neuraminidase [21]. Inhibition of the enzyme prevents the release of virus from the host cell and stops the spread of viral infection. For numerous plant-derived metabolites, the HIV integrase and/or reverse transcriptase (RT) are the targets [22]. The sulfated polysaccharides produced by marine algae belong to the broad-spectrum antivirals (BSAs). They disrupt different phases of viral infection by inhibition of attachment, penetration, uncoating, transcription and translation processes [23]. Cyclosporine A, originally isolated from an ascomycete fungus *Tolypocladium inflatum,* is an approved immunosuppressive drug effective against different viruses. It interacts with cyclophillins and blocks the conformational changes of the viral protein [24,25]. Many other natural antiviral agents have been identified and efforts to discover new bioactive metabolites, increase their efficacy and develop the most cost-effective methods of antiviral drug production are continuing [26,27,28]. 

The aim of the current work was to review the existing knowledge on the antiviral compounds produced by cyanobacteria. These prokaryotic, photosynthesizing microorganisms occur in all types of environments, including seas and oceans, lakes, rivers, hot springs, soil, rocks and ice [29]. They can live in free form or in symbiotic associations with other organisms. Their success in survival and development in different conditions, sometimes at a life limit, can be attributed, among others, to their unique metabolic pathways [29]. According to the latest data, over 2000 cyanobacteria secondary metabolites have been discovered [30]. These include both acute toxins and metabolites that constitute a valuable starting material for the development of novel drugs against cancer, bacterial infections, or metabolic disorders [31,32]. Compared to other activities of cyanometabolites, the antiviral effects have been explored to a lesser extent [32]. Most of the studies were focused on lectins [33] and polysaccharides [34]. 

## 2. Cyanobacterial Lectins

Lectins are monomeric or oligomeric proteins that specifically and reversibly bind to carbohydrates, including those that constitute a component of viral envelope glycoproteins [35]. These compounds are produced by a variety of organisms: plants, animals, fungi and bacteria [36]. Despite some similarity in amino acid sequences, significant differences in their tertiary structure can be observed. The orientation of the carbohydrate-binding domain (CBD) in lectin affects the affinity of the proteins to sugars, mainly the high mannose oligosaccharides, and determines their specificity. The binding potency of lectins is enhanced by the multivalency of CBD [37]. As potent viral entry inhibitors, they can be used in prophylactics, but their role in the treatment of viral infections is also explored [35].

### 2.1. Cyanovirin-N

**Figure 1:**
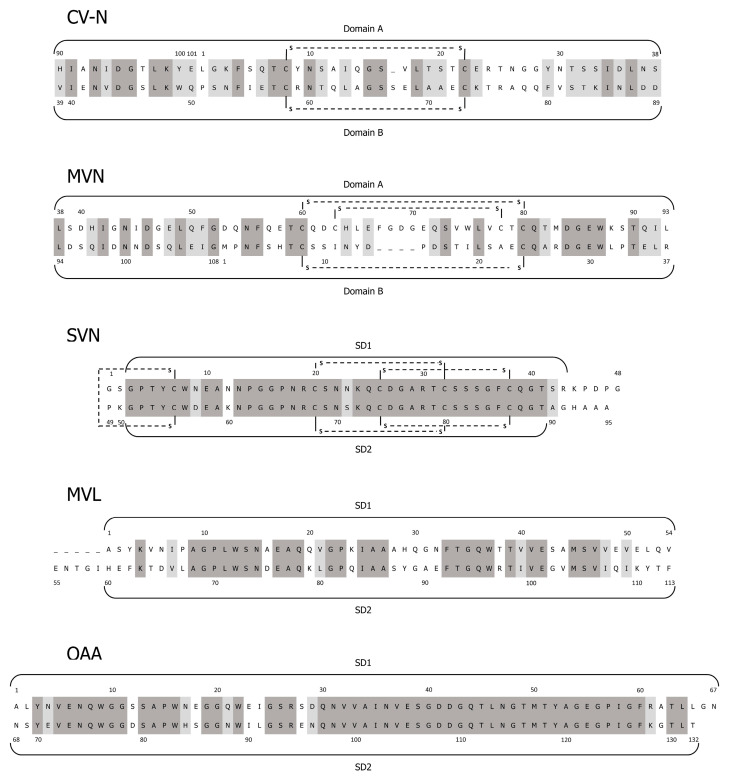
Amino acid sequence alignment of cyanobacterial lectins described in the work: cyanovirin (CV-N), microvirin (MVN), scytovirin (SVN), *Microcystis viridis* lectin (MVL) and *Oscillatoria agardhii* agglutinin (OAA). The structural domains (solid line) and disulfide bonds (dashed line) are marked. Identical residues are indicated in dark grey, and similar residues are in light grey. (The figure was based on the following references: [41] CV-N, [42] MVN, [43] SVN, [44] MVL and [45] OAA).

Cyanovirin-N (CV-N, 11-kDa) was isolated from the culture of the freshwater cyanobacterium *Nostoc ellipsosporum*. The lectin is composed of 101 amino acid residues with a sequence of low homology to other proteins [38,39,40]. In the CV-N structure, two internal repeats containing residues 1–50 and 51–101 of 32% sequence identity were distinguished (Figure 1). 

The fold of the protein is novel, with a three-stranded β sheet structure [40,46,47]. The two carbohydrate-binding domains, A and B, are linked with the four-residue sequence Gln-Pro-Ser-Asn [40,48]. Domain A contains both *N*- (residues 1–38) and *C*- (residues 90–101) termini and is stabilized by a disulfide bond that links Cys58 and Cys73. Domain B occupies the inner part of the protein (residues 39–89) and contains a disulfide bond between Cys8 and Cys22 [39,40] (Figure 1). In solution, CV-N occurs mainly as a monomer, while in crystal, a domain-swapped dimmer is formed [40,46,48]. In the dimmer, the identical (A) or similar (B) domains occur, but they are composed of the sequences derived from two different CV-N monomers [46,48]. CV-N is stable in a broad range of pH and temperature, it is also resistant to organic solvents and detergents [49]. 

**Figure 2:**
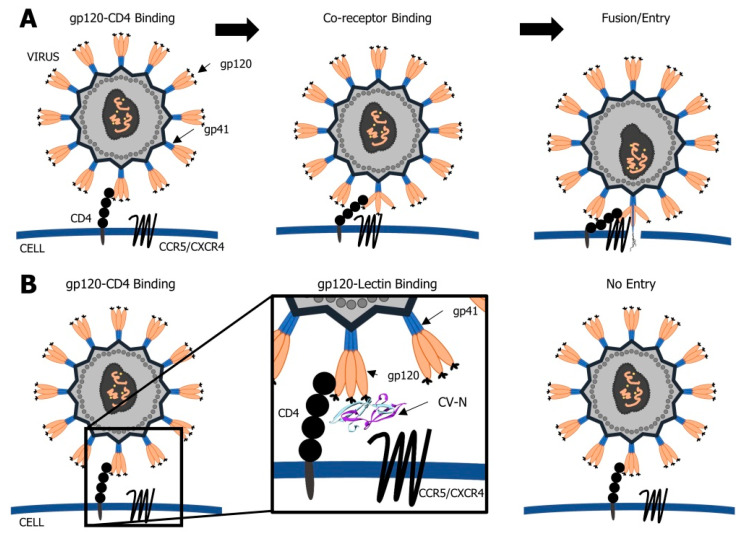
Schematic representation of viral infection (**A**) and the role of cyanobacterial lectin, cyanovirin (CV-N), in inhibition of viral entry and fusion (**B**). CV-N blocks the interaction between the viral gp120 and the CD4 receptor on the host cell. It prevents the interaction with the associated co-receptors CXCR4/CCR5. As a consequence, the virus cannot enter into the cell.

**Figure 3:**
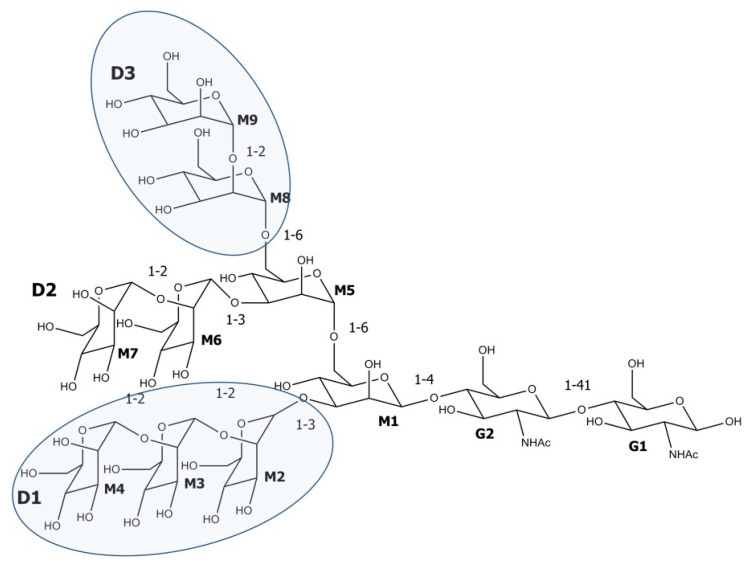
Chemical structure of the high mannose oligosaccharide Man_9_GlcNAc_2_. The cyanovirin (CV-N) recognition sites, Manα(1-2)Man disaccharide units that terminate arms D1 and D3, are marked. Microvirin (MVN) has an affinity for Manα(1-2)Man terminating disaccharide. Scytovirin (SVN) binds to the Manα(1-2)Manα(1-6)Manα(1-6)Man tetrasaccharide core structure, *Microcystis viridis* lectin (MVL) binds to Manα(1-6)Manβ(1-4)GlcNAcβ(1-4)GlcNAc tetrasaccharide and *Oscillatoria agardhii* agglutinin (OAA) recognizes the pentasaccharide sequence Manα(1-3)Manα(1-6)Manβ(1-4)GlcNAcβ(1-4)GlcNAc. M stands for mannose, G for GlcNHAc.

CV-N acts by blocking the interaction between the human immunodeficiency virus HIV gp120 and the CD4 T-cell receptor [48,50]. Binding to viral gp120 prevents the conformational changes of CD4 and interaction with the associated co-receptors CXCR4 and CCR5 (Figure 2). As a consequence, the virus cannot enter into the cell and its transmission from infected to a non-infected cell is blocked. CV-N specifically binds to the terminal Manα(1-2)Man unit of arms D1 and D3 on large high-mannose *N*-linked oligosaccharides (Man8GlcNAc2 (Man8) and Man9GlcNAc2 (Man9)) of HIV gp120 (an epitope of the 2G12 mAb) [38] (Figure 3). In the in vitro studies, the deactivation of HIV was observed at a nanomolar concentration of the lectin [38,51]. The two binding domains of CV-N enable the protein to cross-link the branched oligomannosides and the interaction with both domains is required for the activity of the lectin. However, domain B interacts with α(1-2) linked oligomannose with 10 times higher affinity than domain A. Monovalent mutants of CV-N are inactive [40]. The deletion of *N*-glycans in viral gp120 led to the development of resistance in CV-N exposed strains [52]. 

The potential of CV-N to be developed as a topical microbicide has been extensively explored. Besides interaction with HIV gp120 and inhibition of HIV (type 1 and 2) infection, CV-N is active at a nanomolar level against other enveloped viruses such as simian immunodeficiency virus (SIV) and the chimeric SIV/HIV-1 virus (SHIV89.6P) [53], feline immunodeficiency virus (FIV), human herpes virus 6 (HHV-6), measles virus (MeV) [54], Ebola virus [55], hepatitis virus [56], and influenza virus [57], all with the *N*-linked high mannose oligosaccharides as glycoprotein components. To improve the drug-like properties of CV-N, the protein was modified by site-specific conjugation with polyethylene glycol in a reaction called PEGylation [58]. When administered intravenously, the PEGylated CV-N was significantly less immunogenic.

Due to a broad spectrum of activity against enveloped viruses, and high stability [49], CV-N represents great potential as a prevention measure against viral infections. Therefore, a need to develop alternative or improved methods of CV-N production occurred. Attempts were made to produce recombinant CV-N (rCV-N) at low costs and in amounts sufficient for drug development. The genes involved in CV-N production were expressed in bacterial hosts such as *Streptococcus gordonii* [59], *Escherichia coli* [60], *Lactobacillus jensenii* [61], and in transgenic plants, *Pichia pastoris* and *Nicotiana tabacum* [62,63], reaching the yield of 140 mg/40 g wet cell (40 mg/L) in *E. coli* [47,60]. 

The in vivo tests on simian/HIV (SHIV89.6P) virus-infected macaques (*Macaca fascicularis*), with a rectal and vaginal administration, proved recombinant cyanovirin to be effective in the prevention of sexual transmission of the virus [53]. For these tests, the lectin produced by *E. coli,* with the same activity as natural CV-N, was used. In another study, *L. jensenii* strain 1153-1666 was bioengineered to produce the protein [64]. Repeated vaginal administration of the bacteria in macaques led to a 63% reduction in transmission of SHIVSF_162P3_ and in the peak viral loads. Prolonged production of rCV-N by *L. jensenii* in the vagina did not cause any observable negative effects [64]. When macaques were fed with yoghurt containing the CV-N-producing commensal bacteria (50 mL/day) of the genus *Lactobacillus* (LAB-mCV-N), the lectin was detected in the rectal vault up to ten days after the treatment [65]. In the tests, the 20-fold lower peak of viral infection was observed. Further experiments on macaques showed a positive effect of LAB-mCV-N on the vaginal microbiome [66]. In the case of mice, subcutaneous injection of CV-N (approx. 5 mg/kg) reduced the titers of the Zaire strain of the Ebola virus, but the therapeutic index of the protein was found to be narrow [55]. 

Although the in vivo tests on macaques treated with CV-N did not reveal adverse effects of the lectin, some risk, especially with longer-term usage, still exists. The peripheral blood mononuclear cells (PBMCs) exposed to CV-N showed changes in morphology and were more susceptible to viral infection. In addition, an increase in mitogenic activity and level of chemokines occurred [52,67]. When applied at a 5-fold lower concentration than the antiviral activity range, CV-N enhanced the replication of HIV-1 in PBMCs [52]. CV-N (2 μg/mL) was also toxic to primary human keratinocytes (PHKs) [67].

### 2.2. Microvirin

Microvirin (MVN, 12.7 kDa) was isolated from *Microcystis aeruginosa* PCC7806 [42]. This monomeric protein is composed of 108 amino acids, which form two tandem repeats (residues 1–54 and 55–108) of 35% sequence identity (Figure 1). As in CV-N, these sequential repeats do not correspond to the two structural domains of the protein. Domain A is composed of residues 38–93 and its structure is stabilized by two disulfide bonds linking Cys-60 and Cys-80, and Cys-63 and Cys-78 (Figure 1). The domain contains the only carbohydrate-binding site of the lectin, which interacts with terminal Manα(1-2)Man of viral gp120 glycans [68] (Figure 3). As a monovalent protein, MVN does not cross-link with the branched oligomannosides. In domain B (residues 1–37 and 94–108), there is only one disulfide bond and it links Cys-8 and Cys-24 (Figure 1). 

In vitro tests revealed potent activity of MVN against a wide range of laboratory-adapted, and clinical HIV-1 strains, and also in various cell types (IC_50_ = 2–12 nM) [68,69]. MVN was also found to inhibit syncytium formation between the T-cell line HUT-78 infected by HIV-1 and uninfected HUT-78 cells [69]. The lectin did not inhibit HIV-1 clinical isolates of group O, HIV-2, SIV_mac251_, MLV and the vesicular stomatitis virus (VSV) [47,48]. In contrast to other lectins, including CV-N, MVN had only minor cytotoxic and mitogenic activity; it also did not activate or enhance viral replication in pretreated cells [68,69]. At a dose of up to 7 µM, MVN was not toxic to T-cell line MT-4 and PBMCs. Therefore, it represents a better safety profile than CV-N [69]. In PBMCs, MVN induced the production of several pro-inflammatory cytokines, but with the exception of IL-1B and G-CSF, the effect was less pronounced than in the case of CV-N [69]. 

The MVN resistant virus with mutations in *N*-glycans of gp120 was obtained after 205-day (41 passages) exposure of HIV-1 NL-4.3 to the lectin [69]. The virus also became resistant to carbohydrate-specific human monoclonal antibody mAb 2G12, but was still found to be sensitive to lectins such as CV-N, HHA (*Hippeastrum hybridum* agglutinin), GNA (*Galanthus nivalis* agglutinin) and UDA (*Urtica dioica* agglutinin) [69]. 

As revealed using the HCVcc-Huh-7.5 (hepatitis C virus-human hepatoma 7.5) infection system, both the monomeric and its recombinantly engineered oligomeric MVN forms were active against hepatitis C virus (HCV) [70]. Moreover, the oligomeric variants, especially the trimmers and tetramers, were more potent in neutralizing HIV and HCV than the monomeric MVN. The activity increased with the length of the peptide linker connecting the monomers [70]. LUMS1, the engineered MVN variant composed of two identical domains and with two binding sites, specifically inhibited the infection of HIV-1 and HCV, but was not active against VSV. The potency of HIV-1 (EC_50_ = 37.2 nM) and HCV entry inhibition (EC_50_ = 45.3 nM) by LUMS1 was lower than by MVN (EC_50_ = 8.0 nM) [71]. However, this engineered lectin had a marginal cytotoxic effect on PBMCs, human hepatoma cell line Huh-7.5 and human liver cancer cell line HepG2. LUMS1 also had a negligible effect on the activation of B and T helper (T_h_) cells [71]. 

### 2.3. Scytovirin

Scytovirin (SVN, 9.71 kDa) was isolated from *Scytonema varium* strain HG-24-1 [43]. This single-chained 95-amino acid lectin is composed of two sequence repeats [43,72]. They form two 90% identical structural domains, SD1: 3-43 and SD2: 51-89, separated by a Pro-rich linker [72,73,74] (Figure 1). Each domain contains three aromatic amino acids involved in carbohydrate binding, and two intra-domain disulfide bonds. The fifth inter-domain disulfide bond links Cys-7 and Cys-55 (Figure 1). SVN represents a novel fold, with only short fragments of regular secondary structures and a high number of hydrogen bonds [72,73]. The primary structure of SVN is similar to a chitin-binding group of hevein-like proteins with two chitin-binding sites. However, the arrangements of disulfide bonds and the aromatic triad in the binding site of the proteins are different, which might explain the lack of chitin-binding ability of SVN. The lectin also does not bind to monosaccharides or common trisaccharides [43,72].

SVN binds to the Manα(1-2), Manα(1-6),Manα(1-6)Man tetrasaccharides of the viral enveloped glycoproteins, especially to gp120, but also gp160 and less effectively to gp41 [43]. The binding proceeds simultaneously at the two domains (SD1 and SD2), but domain SD1 has a higher affinity for oligosaccharides than SD2 [75,76]. The in silico studies showed that for the two domains the mechanism of the SVN–Man4 interactions was different [76]. 

SVN possesses potent activity against different HIV-1 isolates (EC_50_ = 0.3–22 nM). The lectin is also active against Zaire Ebola virus (ZEBOV) (EC_50_ 41 nM), Marburg virus (MARV) and HCV (3.2–96 nmol) [77]. In experiments on BALB/c mice, the maximal antiviral protection was achieved when SVN was continually administered before the infection or during the earliest stages of the viral life cycle. Due to low stability in serum, SVN should be dosed every 6 h to be effective [77]. SVN was not toxic to the Huh-7.5.1 cell line at up to 2 µM [78].

The expression of the synthetic gene encoding SVN in *E. coli* yielded 5–10 mg/L of the lectin [75]. The engineered SVN was found to be equally active or showed an even higher affinity to viral glycans [79]. 

### 2.4. Microcystis Viridis Lectin

*M. viridis* lectin (MVL, 13 kDa), a homodimer lectin, was isolated from *M. viridis* NIES-02 [44]. The monomers are composed of 113 amino acid residues, which form two 50% identical domains, *N*-terminal domain (SD1: residues 1–54) and *C*-terminal domain (SD2: residues 60–113), separated by a five-amino acid linker (Figure 1) [80]. Each monomer contains two binding sites, which show affinity to *N*-linked oligomannosides with at least the Manα(1-6)Manβ(1-4)GlcNAcβ(1-4)GlcNAc tetrasaccharide core structure [80,81,82]. The lectin inhibits cell fusion of HIV-1 [81] and HCV [83] with an IC_50_ value of approx. 30 nM. Interestingly, one of the oligomannose binding sites of MVL exerts glucosidase activity and catalyzes the degradation of a chitotriose GlcNAcβ(1-4)GlcNAcβ(1-4)GlcNAcGlcAc_3_ to GlcNAc [82]. It was demonstrated that the antiviral activity of MVL and CV-N is complex and includes both binding of the lectins to the target cell surface and to the viral envelope gp120 [83]. As MVL interacts with cellular proteins, the cytotoxic effects of the lectin might occur. Indeed, the MTT assay revealed an inhibitory effect of the recombinant MVL on Hep-G2 (human hepatocellular liver carcinoma), HT-29 (human colon cancer), SGC-7901 (stomach cancer) and SK-OV-3 (human ovarian cancer) cell lines (IC_50_ 40–53 μg/mL) [84]. This activity could be attributed to the expression of high mannose oligosaccharides during cancer progression [85].

### 2.5. Oscillatoria Agardhii Agglutinin

*O. agardhii* agglutinin (OAA; 13.9 kDa) was isolated from strain NIES-204. This monomeric protein consists of 132 amino acids, which form two 75% identical domains, *N*-terminal domain (SD1: residues 1–67) and *C*-terminal domain (SD2: residues 68–132) [45,86] (Figure 1). The primary structure of the protein was similar to the sequences of hemagglutinin MBHA produced by myxobacterium *Myxococcus xanthus* and the lectin ESA-2 from red algae *Eucheuma serra,* but distinct from lectins produced by *M. aeruginosa* [45,86]. 

OAA possesses two carbohydrate-binding sites located symmetrically at opposite ends of the compound. In contrast to other cyanobacterial lectins (e.g., CV-N, SVN and MVL), which bind to the end mannoses of Man-9, OAA recognizes the branched central core unit of Man-9, a pentasaccharide glycan, Manα(1-3)Manα(1-3)Manα(1-6)Manα(1-6)Man [87].

OAA inhibited HIV replication in MT-4 cells (EC_50_ = 44.5 nM) [45]. Genes encoding lectins and their products with structure, carbohydrate-binding specificity and antiviral activity similar to OAA were discovered in a number of prokaryotic and eukaryotic organisms [45,86,88,89]. This family of lectins was termed *Oscillatoria agardhii* agglutinin homologs (OAAH). They all showed potent antiviral activity against a wide range of HIV-1 and HIV-2 strains and clinical isolates, including HIV-1 group O isolates [90]. OAAH block the viral entry to the target cell and the replication of HIV [86,90]. They also bind to HIV-infected cells with expressed viral glycoproteins on their surface, preventing cell-to-cell transmission of the virus [90]. 

OAA is a stable protein; it preserved its activity even at 80 °C (30 min) and pH 4–11 [86]. The OAA genes were expressed in *E. coli*, and 48 mg/L of the recombinant protein (rOAA) were obtained [88]. Unfortunately, the development of an antiviral agent from OAA might be problematic, because, like CV-N, MVN and MVL, it exerts cytotoxic effects [45]. 

The production of antiviral lectins by cyanobacteria is probably a common feature of these microorganisms. The CV-N homologue, Cyt-CVNH, with approximately 4-fold stronger anti-HIV activity was identified in *Cyanothece* sp. PCC7424 from rice fields in Senegal [91]. Recently, an oscillatorial lectin with anticancer and antiviral activity has been isolated from *Oscillatoria acuminate* MHM-632MK014210 from Egyptian soil habitat [92]. In addition, the genomic screening of cyanobacteria isolated from a lake in the Amazon region led to the identification of new lectins and their homologues [93].

## 3. Cyanobacterial Polysaccharides

Polysaccharides, including chitin, cellulose, glycogen, starch, agar and carrageenan, are the most abundant natural polymers. They constitute structural elements of plants, animals and microorganisms, and play various other roles in their life. Polysaccharides also found wide application in the food industry, cosmetics production, agriculture and medicine [94]. Their anticancer, immunomodulatory, antimicrobial, anticoagulant and wound healing properties have been explored in traditional herbal and modern medicine [95,96]. Polysaccharides of antiviral activity frequently contain sulfate groups and have potent effects on a broad spectrum of viruses, including HIV, HSV, CMV, influenza virus, hepatitis virus and coronavirus [96,97,98,99].

### Calcium Spirulan

Calcium spirulan (Ca-SP), a sulfated polysaccharide, was isolated from *Arthrospira platensis* (previous name *Spirulina platensis*). First, the inhibitory effect of *Arthrospira* water extract on the replication of HSV-1 in HeLa cells (human cervix epithelioid carcinoma) was observed [100,101]. The isolated active agent, Ca-SP, was found to be composed of rhamnose, 3-O-methyl-rhamnose, 2,3-di-O-methyl-rhamnose, 3-O-methylxylose, uronic acids, sulfate groups and calcium ions chelated with sulfate groups [102]. 

In vitro tests showed selective activity of Ca-SP against enveloped viruses, such as HSV-1, HCMV (human cytomegalovirus), MeV (measles virus), MuV (mumps virus), influenza A virus, HHV-6 [101,103], and Kaposi sarcoma-associated herpesvirus/human herpes virus 8 (KSHV/HHV-8) [104]. When added before the infection, Ca-SP reduced viral replication at ED_50_ 0.92–23 µg/mL. In the same experiments, the cytotoxic effects were low; for different cell lines, the ID_50_ ranged from 2900 to 7900 µg/mL [101]. When the quantitative PCR method was applied, the inhibitory activity of Ca-SP against HSV-1 was found to be more potent (IC_50_ 0.05–0.5 µg/mL) and comparable to other antiviral agents such as acyclovir [104]. 

Desulfation or the removal of Ca^2+^ led to the loss of Ca-SP activity. The replacement of the ion with Na^+^ and K^+^ had no significant effects, while the presence of other ions, e.g., Ag^+^ and Cd^2+^, decreased spirulan potency [101,102]. These effects were assigned to the loss of specific spirulan conformation, which was found to be critical for its activity [102].

It is pertinent to note that beside antiviral activity, Ca-SP also induced production of tissue-type plasminogen activator (t-PA) in human fetal lung fibroblasts, showed heparin cofactor II-dependent antithrombin activity [105], and inhibited the invasion and metastasis of tumor cells [106]. 

Other *Arthrospira platensis* metabolites are also active against enveloped viruses such as HIV, HSV and possibly SARS-CoV [107,108]. This microorganism and *Arthrospira*-based products (spirulina extracts) have been used for centuries as protein- and vitamin-rich health food supplements or nutraceuticals. They were shown to have antioxidant, antiviral activity and the ability to boost the immune system. The expanded use of spirulina extracts in food and beverages was approved by the Food and Drug Administration (FDA) [21CFR73.530]. In HIV-patients treated with spirulina extract, a significant decrease in viral load accompanied by an increase in the activity of macrophages, interferon production, NK cytotoxicity and other immune system responses was observed [108,109,110]. In enzymatic hydrolysate from the marine *Spirulina* sp., phycobilin-derived peptides with inhibitory activity against angiotensin-converting enzyme (ACE) were isolated [111,112]. The angiotensin-converting enzyme inhibitors (ACEIs) reduce the production of angiotensin II, responsible for blood vessel constriction. The inhibitors are used in patients with cardiovascular disorders and high blood pressure problems. The inhibition of ACE also enhances the activity of ACE2 (converting angiotensin II into angiotensin), which is downregulated in the SARS-CoV2 infected organs. In COVID-19 patients with severe injury of ACE2 expressing organs, mainly lungs, the therapeutic application of ACEIs has been studied [108]. On the other hand, SARS-CoV2 infection starts with the attachment of the viral spike protein (S-protein) to the host cell ACE2 [113]. Therefore, concerns were expressed that in patients treated with ACEIs, and suffering from cardiovascular disorders, a higher risk of viral infection can occur. Recently conducted clinical trials did not support this hypothesis [114,115]. 

Nostoflan, another antiviral polysaccharide, was isolated from a terrestrial cyanobacterium, *Nostoc flagelliforme* [116]. It contains glucose (42.8%), xylose (29.9%), galactose (20.7%), as well as mannose (6.6%) and glucuronic acid (13.3%) on the nonreducing ends. Unlike many sulfated polysaccharides, nostoflan did not show antithrombin activity [116]. It also has very low cytotoxicity and a wide spectrum of antiviral activity against enveloped viruses such as HSV-1, HSV-2, HCMV, and influenza A virus. When the compound was applied at the onset of the infection, the IC_50_ values for viral replication were 0.37–78 µg/mL [116]. Nostoflan prevented the binding of viral particles to host cells, but did not affect their penetration. Nostoflan also stimulated the response of the immune system in the infected organism [116].

## 4. Antiviral Cyanopeptides and Other Metabolites

**Figure 4:**
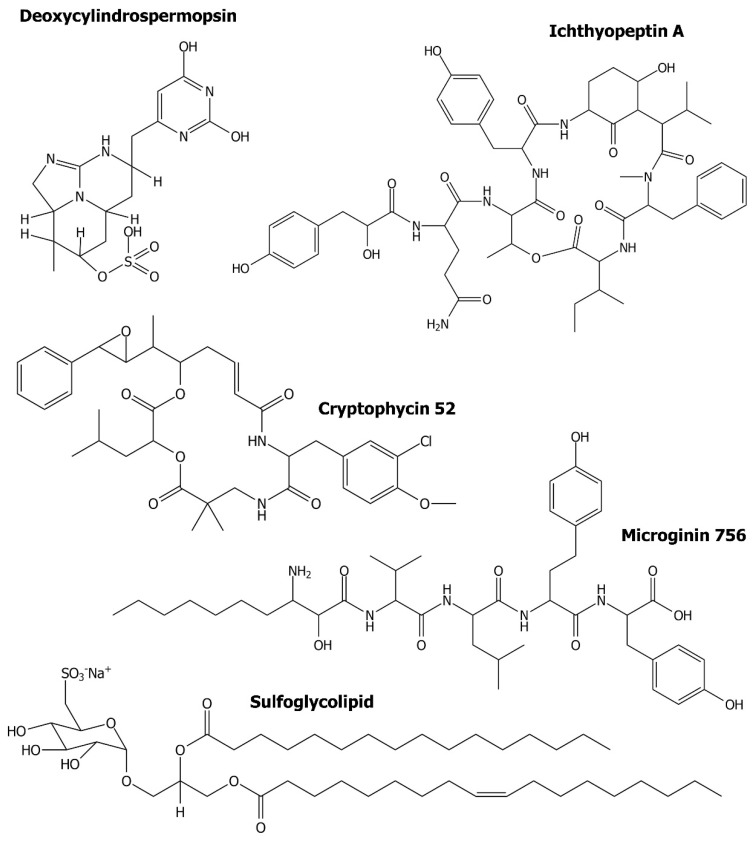
Chemical structure of cyanometabolites discussed in the work as potential antiviral agents.

Peptides constitute another group of antiviral cyanometabolites. A chromatographic fraction containing a mixture of two ichthyopeptins, A and B, inhibited influenza A virus in infected Medin–Darby Canine Kidney cells (MDCK) (IC_50_ = 12.5 µg/mL) [117]. Ichthyopeptins (Figure 4) are cyclic depsipeptides with a unique residue, 3-amino-6-hydroxy-2-piperidone (Ahp), and the general structure PAA-Gln/Asn-[Thr-Tyr/Leu-Ahp-Val/Ile-MePhe-Ile/Val], where PAA is 2-hydroxy-3-(4′-hydroxyphenyl)acetic acid. These peptides were isolated from *Microcystis ichthyoblabe* strain BM Mi/13 [117]. 

The antiviral activity of microginins (MGs) (Figure 4), a class of non-ribosomal peptides produced mainly by the genus *Microcystis*, was not reported. However, their inhibitory activity against ACE [118,119,120] might indicate their potential to prevent SARS-CoV2 infections. MGs contain from 3 to 6 residues and are characterized by the presence of *N*-terminal β-amino-α-hydroxy-decanoic acid (Ahda) or its variants [121,122]. They are also active against aminopeptidases [123,124], but showed no effects against proteases: trypsin, thrombin, plasmin, chymotrypsin, elastase and papain [125]. 

Sulfoglycolipids (Figure 4) were among the first discovered cyanobacterial metabolites with antiviral activity [126]. In these microorganisms, sulfoglycolipids are present in the thylakoid membrane and cell wall of heterocysts. The compounds were isolated from several filamentous species, including *Lyngbya lagerheimii*, *Phormidium tenue*, *Oscillatoria raoi*, *O. trichoides*, *O. limnetica* and *Scytonema* sp. [126,127,128]. Sulfoglycolipids inhibited the DNA polymerase function of the HIV-1 RT with IC_50_ values in the range 24–2950 nM, but had no significant effects on the ribonuclease H [127,128]. The presence of a sulfate group in the sugar unit and the fatty acid ester side chains in the structure was critical for sulfoglycolipid’s activity against HIV RT [128].

The process of antiviral drug development includes both biological tests and in silico studies. The latter ones are performed with the application of structure-based or ligand-based approaches and enable the identification and better understanding of the ligand–target interactions [129]. These techniques reduce the time and costs spent on new drug development and also increase the chances for the design of safe and effective medicine. The in silico techniques were applied in the screening of 23 cyanobacterial metabolites with previously documented anticancer, antimicrobial or antiviral activity [130]. In the study, the molecular docking of the compounds at the binding pockets of two SARS-CoV2 proteases, the main protease M^pro^ and the papain-like protease PL^pro^, was analyzed. These proteases are important targets in antiviral drug development. Of the cyanometabolites, the depsipeptide cryptophycin 52 (Figure 4) and the alkaloid deoxycylindrospermopsin, showed promising effects on the two SARS-CoV2 proteases. The assessment of the physicochemical properties of the compounds performed based on Lipinski’s rule of five [131] led to the conclusion that deoxycylindrospermopsin has the best antiviral drug-like properties. According to Naidoo et al. [130], these results provide a good basis for the development of effective anti-COVID-19 therapy.

In some studies, the potent antiviral activity of crude cyanobacterial extracts was documented, however, the active agents were not identified [132,133,134]. At concentrations non-toxic to MDCK cells, the extracts from several *Microcystis* strains inhibited the replication of influenza A virus [132]. The observed activity was attributed to serine protease inhibitors produced by cyanobacteria [117,132]. The replication of influenza viruses in MDCK cells was also affected by *Leptolyngbya* extracts [133]. The samples inhibited viral neuraminidase, which is one of the antiviral therapy targets [133]. Five other cyanobacterial strains, *Leptolyngbya boryana*, *Arthrospira platensis*, *Nostoc punctiforme*, *Oscillatoria* sp. and *Leptolyngbya* sp., reduced the titers of coxsackievirus B3 (CVB3) in green monkey kidney cell culture and rotavirus (RV) SA-11 in Rhesus monkey kidney cell culture [134]. 

Antiviral activity was found in cyanobacteria representing different taxonomic groups and different metabolite profiles. This indicates that not only cyanobacterial lectins and polysaccharides can be used as starting material for antiviral drug development. Therefore, the efforts to identify the antiviral cyanobacterial products and to determine their pharmaceutical potential are continuing. This is especially important in view of the global and devastating consequences of the COVID-19 pandemic and its negative impact on different areas of our life. The expansion of infectious diseases caused by other viruses also indicates the urgent need for exploration of all potential sources of effective antiviral therapeutics. Considering the increasing threat of pandemic outbreaks caused by different types and strains of viruses as well as their mutation and expansion, the development of broad-spectrum antivirals that do not induce the development of resistance is the biggest challenge and priority.
